# Expanding choice and access in contraception: an assessment of intrauterine contraception policies in low and middle-income countries

**DOI:** 10.1186/s12889-019-8080-7

**Published:** 2019-12-19

**Authors:** Moazzam Ali, Rachel Folz, Madeline Farron

**Affiliations:** 1 Department of Reproductive Health and Research, World Health Organization, Avenue Appia 20, CH-1211 Geneva 27, Switzerland; 2 Division of General Medicine, University of Michigan, 2800 Plymouth Road, NCRC, B16/ 400S03, Ann Arbor, USA

**Keywords:** Intrauterine contraceptive device, LNG-IUS, Long acting reversible contraception, Policies, Family planning

## Abstract

**Background:**

Globally 214 million women of reproductive age in developing regions have unmet needs in modern contraceptives. Intrauterine contraception (IUC) is highly effective, has few medical contraindications, low discontinuation, and is a low cost modern contraceptive method. However, there is relatively low use of IUDs in LMICs. One reason for this may be policies that restrict IUD availability and use. This study assess national policies pertaining to IUD from a diverse set of countries.

**Methods:**

Between December 2015 and February 2016, a 12-question survey pertaining to IUD policy was sent to WHO regional and country representatives.

**Results:**

Sixty-nine surveys were used from countries through WHO regional offices. Among those surveyed, 87% (*n* = 60) had policies pertaining to IUD use. Among them, 84% (*n* = 58) reported that hormonal IUDs were available, but only 42% (*n* = 29) had them in the public sector. Free IUDs in the public sector were available in 75% (*n* = 52) of countries. For IUD promotion, 75% (*n* = 52) of countries reported cooperation with NGOs, and 48% (*n* = 33) received free devices from donors. Policy restrictions beyond the WHO guidelines existed in 15 countries and included restrictions to use for women who were nulliparous, adolescent, unmarried, or had multiple partners.

**Conclusions:**

National policy is important in facilitating modern contraceptive uptake. While many countries who responded in the survey, have policies about IUD use in place, 16% still had none on IUD. Another gap identified was low availability of hormonal IUDs, especially in the public sector. Private sector remains untapped potential in expanding method choice by making IUDs available and accessible in developing countries. Most countries do have policy in place to facilitate IUD use, though there are still gaps in the accessibility of IUDs in many countries. Lastly there is a need to revisit restrictive policies that prevent IUD use for specific populations of women for whom IUDs can be beneficial in realizing their reproductive needs.

## Background

Presently, an estimated 214 million women in low and middle-income countries (LMICs) hope to avoid pregnancy but are not using a modern contraceptive method [1]. While this number has decreased from 225 million women in 2014 as modern method use has increased [1], a large population remains with unmet contraceptive needs. Unmet need for contraception is projected to remain above 10% worldwide through 2030 despite the reductions anticipated for some regions [2]. Understanding the barriers and enablers to unmet contraceptive needs are key to working towards providing contraceptives to those that want them. Of the contraceptives used, intrauterine contraception (IUC) is used by only 13.7% of women (married or in union) and by less than 1 % of women (married or in union) of reproductive age in the least developed countries as of 2015 [3]. Existing data shows great variability between countries and regions [3]. One understudied possible cause for this difference may be policies that impact the availability of intrauterine devices (IUDs), particularly in low and middle-income countries (LMICs) where IUC use is particularly low.

IUDs are highly effective contraceptive devices, working as a long-acting reversible contraception (LARC) option for women. Clinical trials report that IUDs are much more effective at preventing pregnancy than contraceptive pills, patches, or rings [4]. IUDs are also effective for long periods of time and are immediately reversible upon removal. The levonorgestrel or hormonal IUDs (LNG-IUS) are approved for contraceptive use for up to 5 years while the older copper IUDs are rated as effective for 10–12 years [5, 6]. All of these characteristics of IUDs make them an appealing option for those wishing to not become pregnant. In terms of financing family planning programs, IUDs are an extremely cost-effective option [7, 8], as they can be placed and left for several years. Because of the nature of IUD usage, only requiring insertion and removal, less maintenance is required for women and fewer supplies must move through countries’ supply chains as compared to contraceptive pills or injections. Because of these characteristics, IUDs can be very advantageous for family planning programs in LMICs.

Early government policies and pragmatic choices have greatly affected the contraceptive mix available within countries and subsequently the contraception methods women use [9]. In LMICs, funding gaps and donor preferences have especially influenced contraceptive method mix available within countries [10]. International guidelines on IUD usage have also changed over the last several decades, and individual countries’ policies may not reflect these changes [11]. While resources exist regarding contraceptive methods used in countries [3], little is known about the specific policies LMICs have regarding IUDs and their availability in the public and private health sectors. In this study we intend to provide a snapshot of national policies that pertain to IUD use in line with latest WHO recommendations in hopes of comparing policies in countries and addressing areas for improvement in expanding method choice to meet the contraceptive needs of women worldwide.

## Methods

To extract information on IUD policies and availability, a short survey was developed in collaboration with WHO regional advisors and two leading experts in family planning to be disseminated to WHO country representatives to gather information on the country’s IUD policies, government support, and availability of IUDs in the health system. To take advantage of the existing WHO global infrastructure, the data collection was undertaken by contacting WHO regional advisors, who then passed the surveys on to WHO country representatives in their region. The survey included specific questions on governmental policies/guidelines, IUD availability in the public and private sector, cost of IUD in the public sector, Ministry of Health and non-governmental organization (NGO) partnerships surrounding IUDs, if Ministries of Health are obtaining IUDs free from donors (and if so LNG, copper or both), and finally if there are any policies or guidelines that restrict use of IUDs directly or indirectly. There were 12 questions total on the survey (see Additional file 1). Additional space for comments and explanation of previous answers was also provided. The survey was proctored between December 2015 and February 2016. As the survey was optional, some surveys were not returned or were incomplete. When surveys were not returned, three reminders were given. The survey data returned from participating member states were checked for any errors before analysis. Identifying information about the respondent was not required to submit the survey.

A total of 69 usable surveys were received from WHO regional and country representatives (see Table 1).

**Table 1:** Surveys returned by WHO regional office and country

Regional office for Africa (AFRO)	Algeria, Burkina Faso, Burundi, Chad, Congo/Brazzaville, Democratic Republic of the Congo (DRC), Equatorial Guinea, Eritrea, Ethiopia, Gabon, Ghana, Guinea, Madagascar, Malawi, Niger, Nigeria, Senegal, South Africa, South Sudan, Tanzania, The Islamic Republic of Gambia, Togo
Regional office for the Eastern Mediterranean (EMRO)	Bahrain, Djibouti, Egypt, Iran, Iraq, Lebanon, Morocco, Oman, Pakistan, Palestine, Sudan, Syria, United Arab Emirates (UAE), Yemen
Regional office for the Americas (PAHO)	Barbados, Belize, Bolivia, Brazil, Chile, Colombia, Costa Rica, Cuba, Dominican Republic, El Salvador, Guatemala, Nicaragua, Paraguay, Peru, St. Lucia, St. Vincent and the Grenadines, Suriname, Uruguay
Regional office for South-East Asia (SEARO)	Bangladesh, Bhutan, India, Indonesia, Sri Lanka, Timor-Leste
Regional office for the Western Pacific (WPRO)	Brunei, Cambodia, China, Kiribati, Lao PDR, Mongolia, Papua New Guinea, Philippines, Vietnam

Surveys were returned from countries in five out of the six WHO regional offices (Africa, Eastern Mediterranean, Americas, South-East Asia, Western Pacific). These returned surveys provided answers to all survey questions and comprehensive information on the state of IUD availability and restrictions in each country.

## Results

### Existence of national IUD policies

**Fig. 1:**
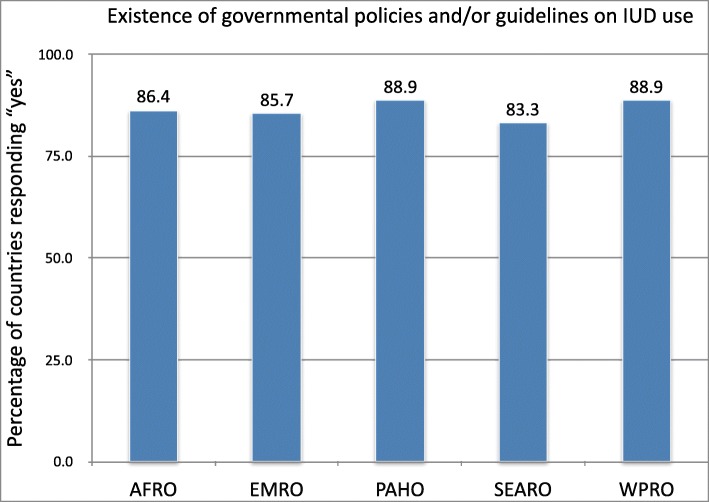
Existence of governmental policies and/or guidelines on IUD use

Among the 69 countries surveyed, 87% (*n* = 60) reportedly included IUDs in official policies and guidelines at the time of the survey, those who did not were South Sudan, Gabon, Eritrea, UAE, Lebanon, Suriname, Barbados, Bhutan, and Brunei. Over the 5 regional offices surveyed (AFRO, EMRO, PAHO, SEARO, WPRO), between 83 and 89% of countries in each region had policy regarding IUD usage in place (see Fig. 1). SEARO had the lowest reported number of countries with IUD policies, while PAHO and WPRO reported the highest.

### LNG-IUS availability through private and public sector

**Fig. 2:**
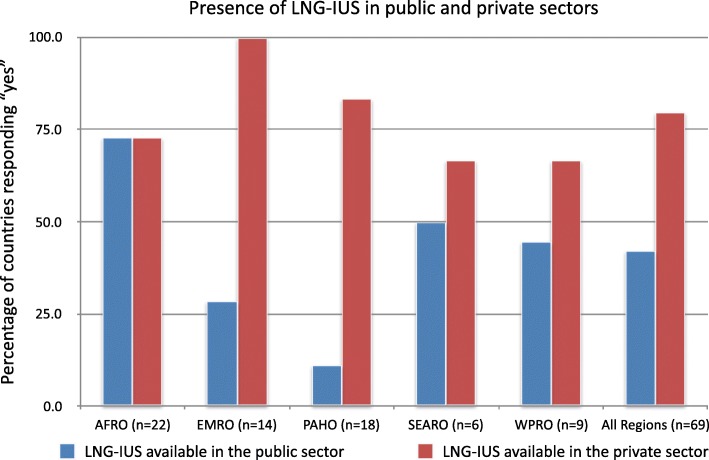
Presence of LNG’IUS in public and private sectors

Of the countries surveyed, 84% (*n* = 58/69) had LNG-IUS available in either the public and/or private sector. The 11 countries that did not report any LNG-IUS available in the public or private section included: Cambodia, Lao, Kiribati, Bangladesh, Cuba, Bolivia, St. Vincent and the Grenadines, South Sudan, Burundi, Congo, DRC. Of the respondent countries, 80% (*n* = 55) answered that LNG-IUS were available in the private sector, and 42% (*n* = 29) respondents answered that LNG-IUS were available in the public sector. Of the regional offices surveyed, the AFRO region had the highest proportion of countries with LNG-IUS available in the public sector (73%), while PAHO had the lowest proportion (11%) (See Fig. 2).

### IUDs provided free of charge in public sector

It was noted that in 75% (*n* = 52) of the respondent countries, IUDs were provided free of charge in the public sector. The countries where IUDs were not provided free of charge in the public sector included: Nigeria, Madagascar, Burkina Faso, Ghana, Togo, South Sudan, Congo/Brazzaville, Senegal, DRC, UAE, Egypt, Suriname, Barbados, Cambodia and Vietnam. Of the regional offices surveyed, the SEARO region had the highest proportion of respondent countries with free IUDs available in the public sector (100%), while AFRO had the lowest proportion (55%).

### Involvement of non-governmental agencies and donors in IUD provision

In the survey, 75% (*n* = 52) of the respondent countries outlined that their national ministry of health was cooperating with NGOs to promote IUD use at the time of the survey. The countries that did not report NGO involvement were South Sudan, Gabon, Eritrea, Sudan, UAE, Oman, Bahrain, Iran, Suriname, St. Lucia, Chile, Paraguay, Brazil, Sri Lanka, Bhutan and Brunei. At the time of the survey, 48% (*n* = 33) of the respondent countries answered that their Ministry of Health received free IUDs from donors. Only 28% (*n* = 19) of surveys answered our survey question regarding IUD type (i.e. copper IUDs or LNG-IUS) provided by donors. Of the 19 respondents, 84% (*n* = 16) received free copper IUDs (Ethiopia, Congo/Brazzaville, Gabon, Senegal, DRC, Syria, Iraq, Palestine, Yemen, Pakistan, Djibouti, St. Lucia, Bolivia, Philippines, Kiribati, Papua New Guinea) and 16% (*n* = 3) received both copper IUDs and LNG-IUS for free (Tanzania, Ghana, Lebanon).

### Policy restrictions on IUD use

Of the 69 respondents, 33% (*n* = 23) of countries reported that there was policy in place to restrict the use of IUDs. 22% (*n* = 15) of these countries listed restrictions beyond the contraindications listed by the WHO’s Medical Eligibility Criteria [11], including: Gambia, Nigeria, Burundi, Congo/Brazzaville, Equatorial Guinea, Malawi, DRC, Morocco, St. Vincent and the Grenadines, Barbados, Belize, Bangladesh, Indonesia, Cambodia and Vietnam. Contraindications listed by the WHO include active STI or PID, current pregnancy, heavy unexplained vaginal bleeding, cervical cancer, or advanced HIV (AIDS) [11].

In addition to WHO guidelines policy, some countries reported IUD restrictions for use by nulliparous women (*n* = 8: Gambia, Burundi, Equatorial Guinea, Morocco, Barbados, Bangladesh, Indonesia, Cambodia), adolescents (*n* = 7: Gambia, Nigeria, Burundi, Congo/Brazzaville, Equatorial Guinea, DRC, Vietnam), women with multiple sexual partners (*n* = 5: Malawi, DRC, St. Vincent & the Grenadines, Belize, Cambodia), and unmarried women (*n* = 3: Nigeria, Indonesia, Vietnam).

## Discussion

This paper reports on the status of policies on IUD in 69 countries. Highly effective LARCs can be an excellent contraceptive choice for clients wishing to avoid unplanned pregnancies. However, IUD’s share of modern method mix is pitifully small, at less than 5%, in 63 countries and only 5–9% in a further 32 countries as noted in a previous study [12]. Countries with especially low IUD use were in Africa [13]. Increasing access to contraceptive devices such as IUDs is a multi-faceted problem that encompasses several factors. In order for a woman to access an IUD, systems must be in place regarding availability of the device, trained provider who can recommend the device, and public and/or private sector support. The woman must also have a positive perception of the device and should be given adequate information about the device and its side effects, which can be fostered by greater availability and knowledge. The wide variability of all of these factors accounts for the broad range of uptake of IUDs globally. It is important for national governments to have policies in place that support women and healthcare providers who want to utilize LARC methods such as IUDs, to make the process of IUD provision as easy as possible.

Several important findings emerge from our analysis. Firstly, this study found that the vast majority (87%) of countries sampled do have policies in place. Despite large variations in IUD usage among the sampled regions, the proportion of countries with policy in place was very similar between all the regions that responded.

While copper IUDs are more widespread and have been available for decades, the newer LNG-IUS devices have a better side effects profile and may become a preferred method among many women globally. It is important to provide women with the option of LNG-IUS for contraception as its availability may increase rates of contraception use. Of the countries sampled, 84% did have LNG-IUS available in either the public, private or both sectors. However, this means that 16% (*n* = 11) of countries sampled are not able to provide women with these devices at all. Only 42% (*n* = 27) countries had LNG-IUS available in the public sector. Moreover, there were large regional variations in the public and private sector availability of LNG-IUS devices. The Africa region reported a roughly even mix of availability in the public and private sectors, while there were large disparities in other regions, particularly PAHO where there was relatively high private sector availability but low public sector availability. The availability of contraceptive devices in the public sector is very important, as this is where the most vulnerable populations receives care. There is a large opportunity here for increased availability of LNG-IUS devices.

Although 75% of the countries surveyed do provide free IUDs in the public sector, however in countries with low public sector IUD availability, there was a possible deficit in IUD utilization as the devices may be out of reach financially. There were also large regional differences in the availability of free IUDs in the public sector with 100% of SEARO countries reporting free IUDs in the public sector and only 55% of AFRO countries reporting free IUD availability in the public sector. These deficits provide opportunities for improvement of IUD availability to women wishing to use these devices and for health systems to provide cost-effective contraception options.

One way to improve accessibility of IUDs is through partnerships with NGOs who not only can aid in promoting the devices, but in many cases can also supply free or subsidized contraceptive devices. At the time of the survey, 75% of the Ministries of Health were working with NGOs on IUD provision and 48% were receiving free IUDs from donors. However, the devices provided were largely copper IUDs, not LNG-IUS devices that may have fewer side effects for some women. These results show that NGOs are greatly aiding in increasing access to IUDs globally. Involvement of NGOs and donations can also aid in reducing costs for women seeking contraception.

An interesting result found in this survey was the variability of restrictions to accessing IUDs imposed on women in the respondent countries and in total 33% of countries surveyed had some sort of policy restriction on IUD use. While some restrictions on IUD provision are medically necessary according to WHO guidelines, such as contraindications to use in the case of active STIs, PID and cervical cancer [11], many countries restrict use beyond these guidelines, targeting specific groups of women. The most common restrictions prohibited adolescents and nulliparous women from obtaining IUDs. Use was also restricted from unmarried women and women with multiple partners. Unfortunately, these populations represent a large number of women who may desire modern contraception and could benefit from using IUDs. Imposing restrictions on these women is medically unnecessary and potentially harmful. These findings highlight the need for countries to revisit their guidelines and policies surrounding the use of IUDs and update them as they see fit as to promote rather than inhibit use of LARC.

### Strengths and limitations

A main limitation of this study is that not all countries responded to the survey and some countries did not complete all questions. The results should therefore be interpreted with caution and should not be extrapolated to other countries that did not respond to the survey. Greater representation from countries would improve the data and help give a clearer picture of global policies surrounding IUD usage. However, we still believe that the response rate provided an adequate geographical spread. Another limitation of this study is that it assesses at formal guidelines and policies of IUD programs in countries, which may not be representative of actual clinical experiences of women or healthcare providers. Informal forces such as stigma or low demand influence IUD usage, and are not reflected in the national policies that were reported. Nonetheless, this snapshot of policies within 69 countries provides useful information about the costs, availability, and restrictions of IUD utilization.

## Conclusions

This survey provided an assessment of the policies surrounding IUD usage and identified gaps in the availability and use of IUC. The results of the survey have shown that while many countries have policy in place to support women in utilizing IUDs, there are several areas for improvement. These include the need for increased access to LNG-IUS in both the public and private sectors, increased access to free IUDs, and the removal of unnecessary restrictions against IUD use in some countries based on nulliparous status, age, marital status, and number of sexual partners. Lastly, the results show the large impact of private sector on increasing access to IUDs globally.

## Supplementary information

**Additional file 1.** Study questionnaire.

## Data Availability

The survey data will be available upon request. Requests can be sent to alimoa@who.int

## Abbreviations

AFRO: Regional office for Africa
AIDS: Acquired immunodeficiency syndrome
DRC: Democratic Republic of the Congo
EMRO: Regional Office for the Eastern Mediterranean
HIV: Human immunodeficiency virus
IUC: Intrauterine contraception
IUD: Intrauterine contraceptive device
Lao PDR: Lao People’s Democratic Republic
LARC: Long acting reversible contraception
LMIC: Low and middle income countries
LNG-IUS: Levonorgestrel-releasing intrauterine system
NGO: Non-governmental organization
PAHO: Regional office for the Americas
PID: Pelvic inflammatory disease
SEARO: Regional Office for South East Asia
STI: Sexually transmitted infection
UAE: United Arab Emirates
WHO: World Health Organization
WPRO: Regional Office for the Western Pacific
