# Supplementary material for: Expanding choice and access in contraception: an assessment of intrauterine contraception policies in low and middle-income countries
Source: BMC Public Health. 2019 Dec 19;19:1707. doi: 10.1186/s12889-019-8080-7 (PMC6924003; doi:10.1186/s12889-019-8080-7)
Supplement: Supplementary file 1 — Additional file 1. Study questionnaire. [file 12889_2019_8080_MOESM1_ESM.docx]

**Intrauterine Conceptive Device (IUD) policy related questionnaire:**

1. Do Governmental policies (and/or guidelines) exist on Intrauterine contraceptive device (IUD) use?

2. If National policy on IUD exists, can you provide the policy details in line with WHO receommndations

3. Are levonorgestrel releasing IUSs available in the public sector? Yes/No

4. Are levonorgestrel releasing IUSs available in the private sector? Yes/No

5. Are IUDs provided free of charge in the public sector? Yes/ No

6. Which categories of staff, apart from medical doctors, are allowed to insert IUDs?

7. To what extent, if any, is immediate postpartum IUD insertion available at hospitals and maternity units?

8. Is the MoH currently cooperating with any NGOs to promote IUD use?

9. Does MoH obtain IUDs free of cost from donors? Just Copper T or LNG also?

10. What is the status of implementation of the above mentioned policies (and/or guidelines)? (At National, state/provincial or subnational levels)

11. Do the policies (and/or guidelines) indicate and place directly or indirectly, any restriction to IUD use? E.g. use by age, parity etc. Kindly provide the details

12. In case, if there is NO explicit National policy (and/or guidelines) on IUD use (or is absent), can you identify and describe the main reasons?. Your perspectives and assessment will be helpful in better understanding of the local policy environment.
